# Assessment and Prediction of Depression and Anxiety Risk Factors in Schoolchildren: Machine Learning Techniques Performance Analysis

**DOI:** 10.2196/32736

**Published:** 2022-08-31

**Authors:** Radwan Qasrawi, Stephanny Paola Vicuna Polo, Diala Abu Al-Halawa, Sameh Hallaq, Ziad Abdeen

**Affiliations:** 1 Department of Computer Science Al-Quds University Ramallah Occupied Palestinian Territory; 2 Department of Computer Engineering Istinye University Istanbul Turkey; 3 Center for Business Innovation and Technology Al-Quds University Jerusalem Occupied Palestinian Territory; 4 Faculty of Medicine Al-Quds University Jerusalem Occupied Palestinian Territory; 5 Al-Quds Bard College for Arts and Sciences Al-Quds University Jerusalem Occupied Palestinian Territory

**Keywords:** machine learning, depression, anxiety, schoolchildren, school-age children, children, youth, young adult, transition-aged youth, early childhood education, prediction, random forest

## Abstract

**Background:**

Depression and anxiety symptoms in early childhood have a major effect on children’s mental health growth and cognitive development. The effect of mental health problems on cognitive development has been studied by researchers for the last 2 decades.

**Objective:**

In this paper, we sought to use machine learning techniques to predict the risk factors associated with schoolchildren’s depression and anxiety.

**Methods:**

The study sample consisted of 3984 students in fifth to ninth grades, aged 10-15 years, studying at public and refugee schools in the West Bank. The data were collected using the health behaviors schoolchildren questionnaire in the 2013-2014 academic year and analyzed using machine learning to predict the risk factors associated with student mental health symptoms. We used 5 machine learning techniques (random forest [RF], neural network, decision tree, support vector machine [SVM], and naive Bayes) for prediction.

**Results:**

The results indicated that the SVM and RF models had the highest accuracy levels for depression (SVM: 92.5%; RF: 76.4%) and anxiety (SVM: 92.4%; RF: 78.6%). Thus, the SVM and RF models had the best performance in classifying and predicting the students’ depression and anxiety. The results showed that school violence and bullying, home violence, academic performance, and family income were the most important factors affecting the depression and anxiety scales.

**Conclusions:**

Overall, machine learning proved to be an efficient tool for identifying and predicting the associated factors that influence student depression and anxiety. The machine learning techniques seem to be a good model for predicting abnormal depression and anxiety symptoms among schoolchildren, so the deployment of machine learning within the school information systems might facilitate the development of health prevention and intervention programs that will enhance students’ mental health and cognitive development.

## Introduction

### Background

Mental health conditions are emerging as health priorities around the globe, with depression being the main cause of illness among adolescents [1], as all other aspects of life in the early adolescent years are greatly affected by poor mental health. The majority of mental health disorders appear by the age of 14 years, yet they may go untreated and thus present severe consequences for the child’s mental, physical, and social health in the long term [2]. The early detection and treatment of mental health conditions in childhood and adolescence not only enhance the child’s quality of life, academic performance, physical health, and social life but also helps them cope with external risk factors as adults [3,4].

With over 300 million official diagnoses worldwide, depression is the most prevalent mental health condition [5]. Although ordinarily and mistakenly interchanged, as well as with a major overlap within their symptoms and treatment, depression and anxiety are 2 distinct diagnoses with different consequences for the patient [6]. The main symptoms of depression include memory loss, lack of concentration, inability to make decisions, loss of interest in daily life activities, feelings of guilt, irritation, and in some cases, suicidal thoughts [6,7]. Anxiety, on the other hand, is an “aversive emotional and motivational state occurring in threatening circumstances” [5], whereby an individual is unable to make decisions or identify the best behavior to remove or instigate the threat. Furthermore, the overlapping depression and anxiety symptoms might affect several of the students’ life areas, including school and family life, friendship, and academic performance. Studies found that depression and anxiety could lead to a lack of attention or motivation, which could influence schoolchildren’s performance [8-11].

Although both anxiety and depression are conditions that severely affect schoolchildren and adolescents and have been shown to predict future mental health problems, interventions are focused on prevention or treatment instead of prediction and risk factors [12].

### Prior Work

Schoolchildren’s mental health problems including depression and anxiety have been studied by many researchers [12-16]. Research studies have reported that depression and anxiety lead to several negative consequences on children’s health development, such as functional impairment and poor cognitive development, social development, and educational and academic performance. It has been found that depression and anxiety symptoms are associated with many risk factors, including poor lifestyle and eating habits, violent behavior, negative social and family support, and socioeconomic factors [13-16].

Data mining and machine learning (ML) techniques have previously been used for mental health prediction, yet most researchers have focused on target populations besides schoolchildren [4,6,17]. Several ML algorithms were used in predicting anxiety- and depression-associated risk factors, such as support vector machine (SVM), convolutional neural network, random forest (RF) tree, and naive Bayes (NB). For instance, Seah and Shim [7] used data mining techniques on social media users, particularly the Reddit platform, to understand the risk factors associated with suicide. Wang et al [18] studied the change of anxiety severity and prevalence among undergraduate students undergoing web-based learning during the COVID-19 pandemic using the XGBoost ML model. Priya et al [4] aimed to predict anxiety, depression, and stress among employed and unemployed individuals through the use of 5 different ML algorithms. Richter et al [6] used ML for differentiating the symptoms of anxiety and depression among adult patients. In 2 studies [4,19], RF and NB algorithms reported an average accuracy rate of 71% and 73% for anxiety and depression symptoms, respectively. Rois et al [20] assessed the performance of different ML algorithms in predicting depression, anxiety, and stress among Bangladeshi university students and found that the RF model had the highest accuracy level of 89.7% and the logistic regression model had the lowest accuracy level of 74.5%. Furthermore, in Priya et al [4], decision tree (DT), NB, SVM, RF, and k-nearest neighbor algorithms were used in predicting anxiety and depression among adults aged 20-60 years, and the study found that the RF algorithm had the highest performance accuracies of 79.8%, 71.4%, and 72.3% for depression, anxiety, and stress, respectively.

Overall, ML techniques have been successfully used as predictors for childhood obesity [21,22], academic performance [23,24], children’s personalities [25], and cognitive performance [1,5,26,27], among others. Thus, the literature has proven that ML is an effective methodology for the prediction of risk factors in several fields—one of which is mental health. The RF classifier and ensemble techniques have been shown to have the highest accuracy rates [4].

### Aim

The role of ML in predicting mental health conditions among schoolchildren specifically has seldom been explored. Therefore, this study aimed to assess the accuracy of 5 ML techniques in predicting depression and anxiety and its associated risk factors (mental health, physical health, social support, and violence, among others) among schoolchildren in Palestine.

## Methods

### Data Collection

The data were collected from the multidisciplinary research project on the Determinates of Students’ Health (physical, mental, and social) in the West Bank and East Jerusalem, conducted in collaboration between the Ministry of Education and Al-Quds University in the 2013-2014 academic year. The study sample included students in fifth to ninth grades (aged 10-15 years) in public schools (administered by the Palestinian Authority) and United Nations Relief and Works Agency schools. A representative sample of 3984 students selected from 100 schools was used in this study.

### Ethics Approval

The study received ethical approval from the Ministry of Education and Al-Quds University Institutional Review Board (05-Aug-2013-12/10).

### Variables

The data set included the associated risk factor variables related to depression, anxiety, physical, and social health, in addition to the sociodemographic variables evidenced in Table 1.

**Table 1:** Machine learning models’ variables.

Variable name	Description	Value
Gender	Gender	Boy or girl
Age	Age	10-15 years
FAS^a^	Economic status	Low, medium, or high
ST	School type	Public or refugee
LP	Living place	Urban or nonurban
FatherEdu	Father’s education	≤Secondary or >secondary
MotherEdu	Mother’s education	≤Secondary or >secondary
HealthyIntake	Healthy food intake	Low or high
UnhealthyIntake	Unhealthy food intake	Low or high
BMI	Body mass index	Normal, overweight, or obese
Smoking	Tobacco risk	Yes or no
PAL	Physical activity	Low active or active
FAL	Free-time activity	Low active or active
FSL	Family support level	Low, moderate, or high
PSL	Peer support level	Low, moderate, or high
SSL	School support level	Low, moderate, or high
PTSD^b^	Posttraumatic stress symptoms level	Low, moderate, or severe
Depression	Depression symptoms	Normal or abnormal
Psychosomatic_SympL	Psychosomatic symptoms	Low, moderate, or severe
Health_Perciption	Positive health perceptions	Low, medium, or high
LSL	Life satisfaction level	Low, medium, or high
Academic_Score	Average grades score	Excellent/very good, good, or weak/fail
Violence	Home violence	Never, sometimes, or often true
Bullying	Bullying behaviors	Never, bullied, or bully/victim
Anxiety	Anxiety symptoms level	Normal or abnormal

^a^FAS: family affluence scale.

^b^PTSD: posttraumatic stress disorder.

#### Sociodemographic Variables

The variables considered were gender, age, degree level, place of residence, household income, school category (public or United Nations Relief and Works Agency), and parents’ education.

#### Depression

The depression data were collected using the 18-item Depression Self-Reported Scale (DSRS) [28] for children aged 8 to 14 years. The DSRS items were composed of 3 answer categories (never=0, sometimes=1, and always=2), with the highest score indicating higher depression. The item “I like to play outside my home” was excluded given that, in Palestinian culture, girls do not go outside for play. The total score was calculated through the addition of the scale items’ answers. The DSRS total score was classified into the following groups:

- Normal: between 0 and 9 points.
- Mild or moderate depression: between 10 and 20 points.
- Severe depression: higher than 20 points.

To improve the performance of ML prediction, the mild or moderate and severe depression categories were grouped into 1 category (called the abnormal category). The final scale was classified into the normal and abnormal categories.

#### Anxiety

The 7-item General Anxiety Disorder [29] scale was used for measuring anxiety. The anxiety score was estimated by assigning the scores of 0, 1, 2, and 3 to the response categories of “not at all,” “about every week,” “more than once a week,” and “every day,” respectively. The scores of 5, 10, and 15 were taken as the cutoff points for mild, moderate, and severe anxiety, respectively. To improve the performance of ML prediction, the mild, moderate, and severe anxiety categories were grouped into 1 category (called the abnormal category). The final scale was classified into the normal and abnormal categories.

#### Physical Activity

The students were asked 3 questions to collect the following data on the levels of physical activity: (1) the number of days the students were physically active for more than 1 hour in the last week, (2) the frequency of the number of hours of playing sports outside of school, and (3) the total number of hours of physical exercise per week.

#### Free-Time Activity

The following data on students’ free-time activities were collected from the students through questions: (1) the daily number of television-watching hours, (2) the number of hours spent on using the internet per week, and (3) the daily number of hours spent on playing video games.

The physical and free-time questions considered the weekdays only (excluding weekends). The activities categorization used the quartiles analysis. For physical activity, the active level of students was identified by the upper quartiles range, whereas the inactive level was identified by the lower quartiles range. For free-time activities, the inactive students were identified by the upper quartiles range, whereas the active ones were identified by the lower quartiles range.

#### Food Intake

The students’ food intake information used the 7-item food frequency scale. The scale items were classified into 7 categories based on intake profile similarity: (1) legumes and vegetables; (2) fruits; (3) milk, yogurt, cheese, and alternatives; (4) sugar; (5) soft drinks; (6) juices and beverages; and (7) energy drinks. The response categories were (1) never, (2) one to two times/week, (3) three to four times/week, and (4) five to seven times/week (almost daily). Students were categorized into 2 groups, healthy and unhealthy intake, based on the item scale sum. The healthy intake group included participants who reported that they did not consume unhealthy food groups (soft drinks, sugar, or energy drinks), and the unhealthy group included participants who did not report to consume healthy food items (vegetables, fruits, milk, and dairy products).

#### Social Support

The variable considered 3 aspects of support: (1) family, (2) schoolteachers, and (3) peers. Each survey section included 2 items related to the students’ communication frequency with the above aspects of social support.

#### Health Perceptions

The perception of health and life was assessed using the 6-item perception scale. The scale items included questions about the students’ self-perception of health and life quality.

#### Life Satisfaction

Students were asked to evaluate their life satisfaction by ranking it from 0 to 10, with 0 indicating the worst life satisfaction and 10 indicating highly satisfied.

#### Posttraumatic Stress Disorder

A 20-item posttraumatic stress symptoms measurement scale was used. The scale measured the anxiety disorder caused by an intensely stressful event. The items were ranked on a 5-point scale from 0, indicating “never,” to 4, indicating “very much.”

#### Academic Performance

This variable was obtained from the students’ academic records. The average performance score was calculated for 6 school subjects: Arabic language, English language, Religion, Social Studies, Science, and Mathematics. The total score was identified by the following categories: excellent/very good, good, or weak/fail.

#### Home Violence

The home violence variable was assessed through 5 items rated on a 3-point scale; the answer options were (1) never, (2) sometimes, or (3) often true. Higher scores point to a higher occurrence of home violence.

#### School Violence and Bullying

These variables were assessed by asking questions related to the frequency of bullying either experienced or witnessed at school. There were 4 violence and bullying categories identified: 0 for not engaged in bullying behavior; 1 for bullying others only; 2 for bullied only; and 3 for bully-victim (those who were both bullies and victims).

#### Psychological Attributes

The parent version of the Strengths and Difficulties Questionnaire was used to assess students’ psychological attributes [30]. The scale was composed of 25 items covering negative and positive attributes; each item was answered with either “not true=0,” “somewhat true=1,” and “certainly true=2.” The scale included emotional symptoms, behavior problems, hyperactivity or inattention, peer relationship problems, and pro-social behavior. In all, 3 categories were identified from this scale: 0 for “normal” (0-13 points), 1 for “borderline” (14-16 points), and 2 for “abnormal difficulties” (≥17 points).

### ML Algorithms

In this study, 5 ML predictive models (artificial neural network [ANN], RF, SVM, NB, and DT) were built and compared to each other to consider their predictive accuracy on the given data set. This predicted the depression and anxiety symptoms among schoolchildren according to the severity level. The data set was divided into the ratio of 70:20:10, representing training, testing, and validation, respectively. The cross-validation approach with grid search method was used for parameters optimizations. The parameters for different algorithms were set as follows:

- In the ANN model, the hidden layer had 500 neurons, with 500 as the maximum number of iterations based on logistic activation function.
- The RF model had 1000 trees and 5 maximum depth trees. The maximum number of samples for splitting the internal nodes was set to 2, and the leaf node minimum number was set to 1.
- The SVM regularization parameter was set to 20, the Radical Basis Function kernel was set to 0.001, and the bias error control factor was set to 1.

Based on the optimization results, the algorithms were used in predicting the depression and anxiety symptoms. The ML algorithms used are described in Table 2.

**Table 2:** Description of machine learning techniques.

Machine learning algorithm	Description
Artificial neural network	Neural networks are a series of algorithms that recognizes relationships between sets of data. The algorithm is built of many small, classified aggregators that feed-forward from the input data to the target prediction [31,32].
Random forest	Random forest is an ensemble learning technique that is used for classification along with regression through decision trees and outputs the plurality of votes from the trees [33]. Each tree is exposed to a data subset and independently evaluates the features available to arrive at a conclusion [34,35].
Support vector machine	The support vector machine is an algorithm used for classification in addition to regression analysis. Support vector machine creates a decision surface for the prediction of variables starting from a small number of similar cases across the support vector and then classifying the remaining cases based on how they fall on the side of the support vector [34,35].
Decision tree	A decision tree is an algorithm that builds a tree-like structure for classifying features with multiple levels of observations [32]. The substructures, “leaves,” represent the objects’ class, whereas the “branches” represent the features [34-36].
Naive Bayes classification	Naive Bayes is the easiest and most powerful algorithm to predict features within a data set. This machine learning algorithm analyzes the training sets across the variables to find how likely the variables’ ability is for predicting the target [34,35].

### Data Analysis

The data variables were cleaned and normalized before analysis. The data set consisted of 3984 student records. The ML algorithms were applied to predict the students’ mental health depression and anxiety symptoms by using the Orange data mining software [37] for testing and validating the ML models.

Different performance measures were used to evaluate whether the ML models can predict schoolchildren’s depression and anxiety symptoms, such as accuracy, specificity, precision, recall, and F-measure. The calculating equations for performance measure are as follows: 

Specificity = True Negative / (False Positive + True Negative) **(1)**

Precision = True Positive / (True Positive + False Positive) **(2)**

Recall = True Positive / True Positive + False Negative **(3)**

F-measure = (2 × Precision × Recall) / (Precision + Recall) **(4)**

Accuracy = (True Positive + True Negative) / (True Positive + True Negative + False Positive + False Negative) **(5)**

## Results

A brief descriptive analysis was performed to present the depression and anxiety levels among the Palestinian schoolchildren and understand the data distribution before the evaluation of ML techniques. The data set was composed of 3984 students with a mean age of 13 (SD 1.5) years, ranging from ages 10-15 years. Among these students, approximately 29.8% (n=1188) were boys and 70.2% (n=2796) were girls. Data in Table 3 show the depression levels distributed by grade levels. The eighth and ninth grades students reported the highest moderate depression levels (eighth: 61%, 469/769; ninth: 61.5%, 494/803). The seventh and ninth grades students reported the highest severe depression levels (seventh: 6.6%, 54/824; ninth: 7.3%, 59/803). The results show that more than half (57.3%, 2283/3984) of the students reported a moderate level of depression in all ages.

**Table 3:** Students’ depression and anxiety levels by grade.

Mental health condition, grade		Normal, n (%)	Moderate, n (%)	Severe, n (%)
**Depression**				
	Fifth grade (N=797)	362 (45.4)	404 (50.7)	31 (3.9)
	Sixth grade (N=791)	288 (36.4)	460 (58.2)	43 (5.4)
	Seventh grade (N=824)	314 (38.1)	456 (55.3)	54 (6.6)
	Eighth grade (N=769)	256 (33.3)	469 (61)	44 (5.7)
	Ninth grade (N=803)	250 (31.1)	494 (61.5)	59 (7.3)
**Anxiety**				
	Fifth grade (N=797)	452 (56.7)	147 (18.4)	198 (24.8)
	Sixth grade (N=791)	426 (53.9)	164 (20.7)	201 (25.4)
	Seventh grade (N=824)	432 (52.4)	203 (24.6)	189 (22.9)
	Eighth grade (N=769)	438 (57)	172 (22.4)	159 (20.7)
	Ninth grade (N=803)	479 (59.7)	192 (23.8)	133 (16.6)

The results in Table 3 show the percentage distribution of anxiety levels by grade levels. The participants reported a decrease in anxiety severity levels as grade levels increased. Students in the fifth and sixth grades had the highest anxiety levels (24.8%, 198/797 and 25.4%, 201/791, respectively). Overall, more than half (55.9%, 2227/3984) of the students reported normal anxiety levels at all ages. However, the minimum anxiety level found in ninth graders (16.6%, 133/803) might still affect the students’ growth and development. We observed significant differences between the depression and anxiety scores, grades, and genders. The results also indicated that girls reported higher depression (6.4%, 180/2796) and anxiety (22.9%, 640/2796) levels than boys (4.3%, 51/1188 and 20.2%, 240/1188, respectively). Furthermore, the results in Table 4 show that the univariate analysis of depression symptoms indicated a high significant association with posttraumatic stress disorder (PTSD), life satisfaction, health perception, gender, physical activity, family support, smoking, home violence, and grade; whereas for anxiety, the results show a significant association with PTSD, family support, school support, friend support, grade, home violence, gender, and bullying behaviors.

Table 5 demonstrates the comparison between ML algorithms' accuracy rates for the models used in predicting students’ depression and anxiety. Besides the SVM and RF models, which had the highest accuracy rates (depression: 92.6% and 92.4%, respectively; anxiety: 76.5% and 78.4%, respectively), the other ML algorithms had acceptable performance accuracies. The 2 classes of depression and anxiety resulted in the confusion matrix depicted in Table 6; the columns show the predicted classes, whereas the rows show the actual classes. To further present prediction accuracy, the instances classification accuracy of the 5 models is shown in Table 7.

**Table 4:** The univariate analysis of depression and anxiety symptoms by study variables.

Mental health condition, variable		*F* test (*df*)	*P* value
**Depression**			
	PTSD^a^	643.5 (1,3983)	<.001
	Life satisfaction	83.6 (1,3983)	<.001
	Positive health perception	34.6 (1,3983)	<.001
	Gender	12.5 (1,3983)	<.001
	Physical activity	11.1 (1,3983)	<.001
	Family support	9.4 (1,3983)	<.001
	Smoking	7.5 (1,3983)	.006
	Grade	5.7 (1,3983)	<.001
**Anxiety**			
	PTSD	105.2 (1,3983)	<.001
	Family support	59.5 (1,3983)	<.001
	School support	46.0 (1,3983)	<.001
	Friend support	24.4 (1,3983)	<.001
	Grade	6.1 (1,3983)	<.001
	Home violence	6.0 (1,3983)	.002
	Gender	5.5 (1,3983)	.02
	Bullying behaviors	5.0 (1,3983)	.001

^a^PTSD: posttraumatic stress disorder.

**Table 5:** Comparison of prediction accuracy among the 5 machine learning models.

Model	Depression, %	Anxiety, %
Decision tree	88.5	74.1
Support vector machine	92.6	76.5
Random forest	92.4	78.4
Artificial neural network	91.9	75.7
Naive Bayes	87.1	72.7

**Table 6:** Confusion matrix of the machine learning models’ performance.

Machine learning algorithm, actual		Depression, predicted		Anxiety, predicted	
		Normal	Abnormal	Normal	Abnormal
**Decision tree**					
	Normal	3200	293	2752	590
	Abnormal	360	1832	864	1479
**Random forest**					
	Normal	3096	397	2856	486
	Abnormal	37	2155	730	1613
**Naive Bayes**					
	Normal	2766	727	2713	629
	Abnormal	18	2174	905	1438
**Support vector machine**					
	Normal	3068	425	2570	772
	Abnormal	2	2190	570	1773
**Neural network**					
	Normal	3141	352	2746	596
	Abnormal	111	2081	775	1568

**Table 7:** Performance measures analysis for the different machine learning models.

Model, mental Health condition		AUC^a^, %	CA^b^, %	Error rate, %	F1-score^c^, %	Precision, %	Recall, %
**Decision tree**							
	Depression	86.7	88.5	88.5	88.5	88.5	86.7
	Anxiety	73.7	74.4	74.1	74.2	74.4	73.7
**Support vector machine**							
	Depression	96.8	92.5	92.6	93.7	92.5	96.8
	Anxiety	82.1	76.4	76.5	76.8	76.4	82.1
**Random forest**							
	Depression	97.2	92.4	92.4	93.3	92.4	97.2
	Anxiety	86.8	78.6	78.4	78.5	78.6	86.8
**Artificial neural network**							
	Depression	96.8	91.9	91.9	92.3	91.9	96.8
	Anxiety	84	75.9	75.7	75.7	75.9	84
**Naive Bayes**							
	Depression	95.5	86.9	87.1	89.9	86.9	95.5
	Anxiety	82.3	73	72.7	72.8	73	82.3

^a^AUC: area under curve.

^b^CA: correspondence analysis.

^c^F1-score: harmonic mean between precision and recall.

Table 7 shows the different performance measures (area under curve [AUC], accuracy, error rate, F1-score, precision, and recall) calculated for the 5 ML models. The results in Table 7 indicated that the highest accuracy rates for both variables, depression and anxiety, was achieved by the SVM and RF algorithms. Nevertheless, the results of the confusion matrix in Table 6 show imbalanced classes of depression and anxiety classifications by the ML algorithms, which means that accuracy measure will not provide sufficient performance measure. Therefore, we used the harmonic mean of recall and precision (F1-score) as an additional performance measure for the selected ML algorithms. The F1-scores obtained by the SVM and RF models were the highest for both depression and anxiety, whereas the NB model reported the lowest accuracy and F1-scores for both depression and anxiety. TThe classification results show that the RF, SVM, and ANN models presented the highest accuracy levels for predicting students’ depression and anxiety. However, all algorithms used in this study produced an acceptable performance measure for depression and anxiety symptoms.

**Figure 1:**
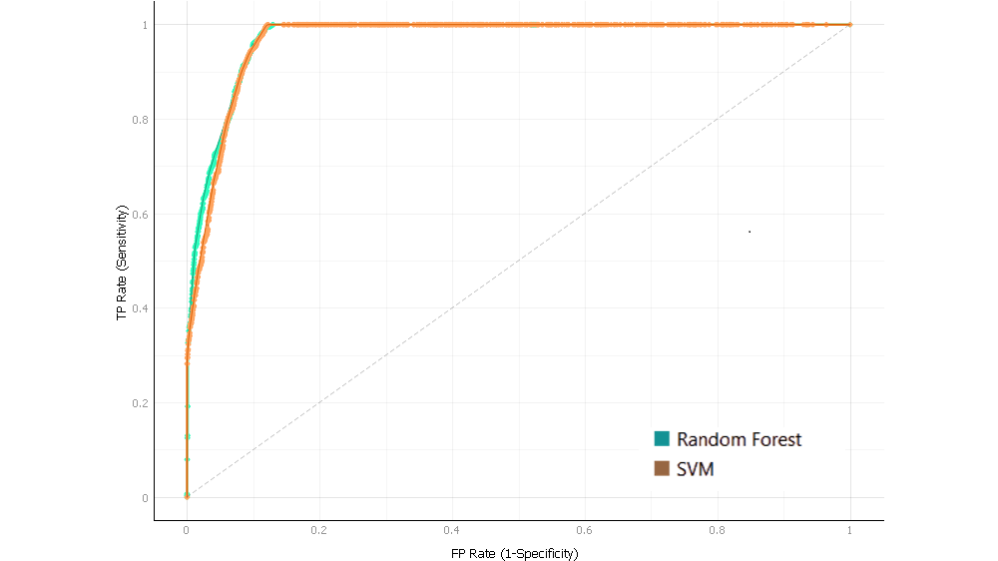
SVM and random forest receiver operating characteristics curve for abnormal depression (TP rate of sensitivity against FP rate of specificity). FP: false positive; SVM: support vector machine; TP: true positive.

**Figure 2:**
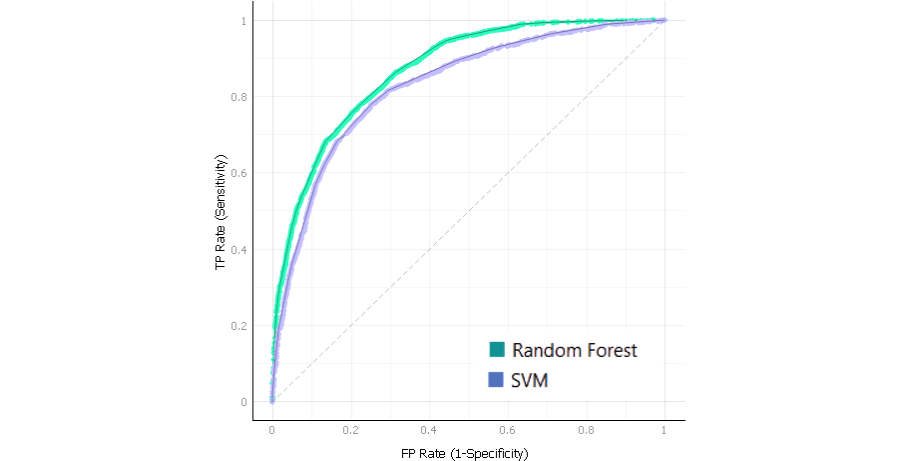
SVM and random forest receiver operating characteristics curve for abnormal anxiety (TP rate of sensitivity against FP rate of specificity). FP: false positive; SVM: support vector machine; TP: true positive.

The RF receiver operating characteristics for the 2 depression and anxiety classes are presented in Figures 1 and 2 and Table 8. There were 2 numerical categories of student depression and anxiety used: normal and abnormal. The receiver operating characteristics resides in the upper left corner; thus, the RF algorithm has a better prediction of positive value than the other studied algorithms (AUC of 82% and 81% for depression and anxiety, respectively).

The features importance ranking for depression and anxiety symptoms were analyzed using the RF ranking method. The features importance was scaled from 0% to 100%. The features with importance greater than 60% were selected and presented in Table 8. The most important features that affected depression symptoms were age, bullying behaviors, PTSD, life satisfaction, anxiety symptoms, health perception, friend support, academic score, school support, home violence, and family income. Psychosomatic symptoms, age, bullying behaviors, family support, PTSD, depression symptoms, friend support, school support, home violence, academic score, family income, and physical activity were the most important features that affected the schoolchildren’s anxiety symptoms.

Other variables were found to be less important for depression and anxiety among schoolchildren and therefore were not presented in Table 8.

**Table 8:** Depression and anxiety symptoms predictors importance ranking.

Mental health condition, symptom		Importance, %
**Depression**		
	Family income	60
	Home violence	66
	School support	71
	Family support	75
	Academic score	78
	Friend support	80
	Health perception	83
	Anxiety symptoms	84
	Life satisfaction	85
	PTSD^a^	90
	Bullying behaviors	94
	Age	95
**Anxiety**		
	Physical activity	60
	Family income	64
	Academic score	70
	Home violence	77
	School support	78
	Friend support	79
	PTSD	83
	Depression symptoms	84
	Family support	88
	Bullying behaviors	90
	Age	93
	Psychosomatic symptoms	96

^a^PTSD: posttraumatic stress disorder.

## Discussion

### Principal Findings and Comparisons With Previous Work

In this study, we used ML models in predicting depression and anxiety symptoms among schoolchildren. The study found that two-thirds of students reported moderate depression symptoms and about 7% had severe depression, whereas around 22% of students reported moderate and severe anxiety symptoms. The data showed that students in the sixth, eighth, and ninth grades had higher depression symptoms, whereas students in the fifth and sixth grades reported higher anxiety symptoms. These results are consistent with similar studies that found high depression and anxiety rates among adolescents [4,15,38-40]. The severe depression level of our study was close to those reported among schoolchildren in Germany, Canada, and Jordan [14,39,41]. The severe anxiety level was consistent with the results reported among adolescents living in Jordan, Spain, Canada, and Saudi Arabia [38,39,41,42].

The performance of the ML algorithms in predicting schoolchildren’s depression and anxiety was assessed using the AUC, accuracy, precision, recall, and F1-score performance measures. In total, 23 relevant features were used after performing feature selection using ML algorithms. The features were used as input variables, whereas the average depression and anxiety scores were considered as the target variables independently. Among the tested models, SVM and RF showed the best performance results for depression (SVM: 92.5%; RF: 76.4%) and anxiety (SVM: 92.4%; RF: 78.6%). Furthermore, the specificity for the SVM and RF models were 87.8% and 88.6% for depression, respectively, and 76.9% and 85.5% for anxiety, respectively. Of the 23 features selected, 16 features were correlated to instances of depression and anxiety, including physical, mental, and social health indicators. However, the other models showed acceptable performance scores in predicting depression and anxiety. Thus, the findings of our study are consistent with other studies that assessed ML models in predicting depression and anxiety among adolescents and adults [4,6,17,43-48]. In Priya et al [4], the NB model had the highest accuracy levels for anxiety, depression, and stress, whereas the F1-score showed that the RF model had the highest performance measure for stress symptoms. Furthermore, the results are consistent with other studies that assessed the ML models in predicting depression and anxiety among adults [44-46]. The studies showed that the ML models are efficient in predicting depression and anxiety symptoms.

Significant risk factors for schoolchildren’s depression and anxiety were found. Poor family and school conditions, such as low levels of school and family support, home and school violence (bullying behaviors), and low levels of positive health perception were highly significant to the risk of suffering from severe and moderate depression and anxiety, mainly among boys. This has been observed through the implementation of ML models. Additionally, the same results were obtained when controlling and not controlling for school type, age, and place of residence.

Furthermore, the data have shown that health-associated factors also had a significant effect on students’ growth, cognitive development, and academic performance [49]. Moreover, it has been found that negative health conditions, such as obesity and PTSD, had a direct negative impact on student's mental health and cognitive development. Conversely, mother’s education, gender, age, locality, physical activity, and good nutrition had less significant effects on mental health issues than the abovementioned variables.

These findings are consistent with other related studies that have found a strong association between mental health problems and risk factors such as school and home violence or negative health conditions [3,43,50,51]. Similar to previous studies, the research has shown that specific conditions such as obesity and PTSD are significantly correlated to depression and anxiety [5,26,52]. Furthermore, the prediction accuracy results obtained from the implemented ML algorithms are equivalent to the prediction accuracy rates obtained from related studies in the fields of mental health, as the RF model proved to be the most significantly accurate model [4,53].

Moreover, this study shows that several ML algorithms can predict depression and anxiety and their associated risk factors. The used algorithms successfully managed to predict the target variables, and the NB algorithm had the lowest accuracy rate for both anxiety and depression. However, it could be considered for the prediction of mental health conditions among schoolchildren with the presented variables. The RF ML model, on the other hand, proved the most effective in predicting depression and anxiety when students’ health (physical and social) and related risk factors are considered. Overall, the classification accuracies were all at a favorable level. These findings show the importance of integrating ML techniques in the fields of mental health. These findings are consistent with other studies that indicated the importance of using ML in psychiatric and mental health diagnosis [4,54,55]. In the study of Haque et al [56], the RF model reported the highest accuracy among other ML algorithms (RF, XGBoost, and DT) in detecting depression among children aged 4-17 years. Furthermore, Sau et al [57] assessed 5 ML algorithms (logistic regression, NB, RF, SVM, and CatBoost) in identifying risk factors associated with anxiety symptoms, with the CatBoost model reporting the highest accuracy among the other ML models.

In this study, the risk features importance rating for anxiety and depression was estimated, which showed that age, bullying behaviors, PTSD, life satisfaction, and anxiety are the 5 most important features in predicting depression symptoms, whereas psychosomatic symptoms, age, bullying behaviors, and depression symptoms are the most important features in predicting anxiety symptoms. The study findings are consistent with other studies that found that the children’s age, academic score, family income, social and family support, school and home violence, and physical activities are significant and important factors in predicting schoolchildren’s depression and anxiety [13,15]. An important contribution of this study is the classification of schoolchildren at high risk to develop anxiety and depression symptoms. The most important features in our model are consistent with previous studies, which found that the population with high risks of anxiety and depression has a higher rate of tobacco use, increased BMI, and decreased academic performance [21,58-60].

### Strengths and Limitations

Currently, the standard mental assessment scales are used in detecting schoolchildren’s depression and anxiety, and it is mainly based on the health care system screening programs, which are mainly used when abnormal symptoms are witnessed. Furthermore, the current practices might fail in detecting the main associated factors with a subsequent delay in detection and intervention. Our prediction model combined the different levels of risk factors, including physical, mental, and sociodemographic factors. Our model is less dependent on the schoolchildren’s subjective awareness of health status and health care screening behaviors; thus, the model improved the automatic and early detection of schoolchildren’s depression and anxiety.

Overall, our study strengthens the need for the implementation and deployment of ML in addressing mental health problems. The early detection and prediction of risk factors associated with mental health symptoms (depression and anxiety) can enhance the development of intervention and prevention programs that improve children’s growth and cognitive development. Thus, this research study not only introduces the ML techniques in predicting depression and anxiety but also provides the policy makers with the power of ML in the early prediction and diagnosis of schoolchildren’s mental health problems.

The study is limited by the number of variables. Based on the findings presented in this paper, future research will benefit from expanding the study by adding additional associated factors, including cognitive development skills, in-school student behavior, social activities, and digital media activities. Furthermore, variables related to external factors, such as the incidence of violence in the community, presence of soldiers, checkpoints, and mobility restrictions are not present in this study, yet these variables would be very relevant to further investigate the risk factors associated with anxiety and depression among schoolchildren in the Palestinian context. The presence of these variables would further enhance the accuracy of the ML prediction models for anxiety and depression.

### Conclusions

The study assessed the accuracy and performance of 5 ML models in predicting the associated health factors on Palestinian schoolchildren’s depression and anxiety. Based on the results presented, this research concludes that ML algorithms, particularly (but not exclusively) RF and neural network, are effective predictive models for students’ mental health status. These models could be integrated into schools’ information systems for the automatic prediction of students’ depression and anxiety based on key features. In this manner, students, families, school staff, and administration will be able to tackle issues that might affect students’ mental health using the obtained prediction results. Likewise, by making use of accurate ML techniques, such as RF, public health professionals, health care providers, and decision makers will be able to predict rising issues and implement relevant intervention programs to enhance students’ health, education, and well-being. 

## Abbreviations

ANN: artificial neural networks
AUC: area under curve
DSRS: 18-item Depression Self-Reported Scale
DT: decision tree
ML: machine learning
PTSD: posttraumatic stress disorder
RF: random forest
SVM: support vector machine

## Data Availability

The data sets generated and/or analyzed during the current study are available from the corresponding author on reasonable request.
